# Her Heart's Mistake

**Published:** 1889-08-03

**Authors:** E. D. Cumming

**Affiliations:** Author of "An Ordeal by Silence"


### Her Heart's Mistake.

By E. D. Cumming, Author of "An Ordeal by Silence."

*(Continued from page 268.)*

### Chapter XVI.—A Step Nearer.

Richard Warren did not go to the club that evening after he parted with Kate Harrison; he walked slowly down the Mall to his own rooms in the compound, and shut the door to prevent friends disturbing him. He threw himself into a chair, and for a full hour sat in silence, pondering over the position into which he had almost unconsciously drifted, past hope of extrication. He loved this girl with all the strength of his nature, and she was engaged to marry another man. He had only fully awakened to the knowledge of his love on that night when she playfully told him that her affianced husband had written chiding her for the intimacy which she had allowed to grow up between them. And he felt that Captain Bairdsley had reason for his letter; not that Kate had ever spoken a word or given a look upon which the most exacting lover could have founded complaint; she had never for a moment behaved as though she reciprocated his feeling in the faintest degree, but Warren felt that there was danger, and very real danger, too. He was sure that she had not yet discovered it, for he knew had she suspected its existence she would never have asked him to renew his frequent informal visits to her father. He was not one of those men who believe that because they love they must be loved in return. He did not imagine that Kate cared for him; but he knew that she liked him sincerely and took pleasure in his society. They understood each other so thoroughly; their deepest sympathies harmonised, and drew them more closely together every day they met and talked. Therein lay the danger. They could not maintain their intercourse much longer before the light broke in upon Kate as it had already broken in upon him. It was impossible that they should meet almost every day and not draw steadily and surely nearer to each other, until he betrayed himself, or until she discovered that her heart had been false to her. He saw whither they were drifting, and he must steer into safer waters before it was too late. He could not avoid meeting her constantly; the groove of Indian life under ordinary circumstances forced them into one another's society. But now, whilst mourning kept her more secluded, he could at least abstain from going to their house; from going out of his way to see her; and since he had realised his love he had not been near the Doctor's bungalow. But now she had asked him to come again, and unless he told her the truth, he must go. What was he to do? In justice to the absent lover he was bound to bring this intimacy to an end, and at once.

He was in his place at the club dinner-table that evening, but his gravity and pre-occupation drew down upon him the good humoured badinage of his companions. He was not a talkative man at any time, but they had never seen him so silent and depressed as he was to-night, and they rallied upon the serious demeanour which contrasted so strongly with the light-hearted gaiety prevalent around him.

"Why is our Warren thus so sad?" enquired Mr. Rendell, the assistant collector of Meerut. "Is he also about to join the ranks of the Benedicts? Has Bairdsley's example led another good man astray?"

"Not yet," answered Warren, with a forced smile.

"I'm glad to hear it," said the other. "I was beginning to fear that Bairdsley was the first victim of a matrimonial epidemic, which might in time leave Dr. Anson and my humble self to support the honour of the club as a dining institution."

"I'm not going to desert you, Rendell; but you might encourage us to remain single if, in your secretarial capacity, you remonstrated with the club cook. He grows worse every day."

"My judicial capacity as Assistant-Collector and my respect for the far-reaching provisions of the Indian Penal Code restrain my avenging hand. We must wait for Bairdsley."

Captain Bairdsley's summary treatment of his servants had gained him a good deal of notoriety in the station, and anyone who complained of his attendant's misconduct was, as a matter of course, recommended to "send for Bairdsley."

"Coming in for a pool to-night, Warren?"

"No, thanks; I have some letters to write for the mail."

The other men adjourned to the billiard-room, and Warren strolled over to his quarters, which were contained in the long range of chambers which stood at right angles to the main building of the club. They were not so large as he could have wished, consisting only of a bedroom and bathroom; but as the former gave space for his writing-table and books, he had at least a refuge when he wanted quietude as he did to-night. He would have preferred a bungalow to himself, but his limited income compelled him to adopt the most economical method of living, and that was to be found at the club. His share of Mr. Pringle's business was bringing in between four and five hundred rupees a month, and promised better things in the future; so he had made himself as comfortable as he could for the time being, and lived in hopes of setting up an establishment of his own by-and-by.

It was a lovely starlight night, and he paced up and down the walk before his rooms, with his hands behind him, thinking over the question he had set himself regarding Kate Harrison. The flying foxes were flapping and screaming in the mangoes overhead, and large light-winged bats were flitting noiselessly around him, swooping and diving in pursuit of their insect prey. A pariah dog sneaked stealthily through the bushes, hurrying away from the terriers, whose excited barking in their owner's rooms warned him that his presence had been discovered. From the distant maidan, the weird shrieking of the jackals betrayed that they had found a carcase for their midnight meal. The discordant braying of horns and the beating of tom-toms in the bazaar, told that a native "tumasha" was in progress; but the thousand noises of the Indian night fell unheeded on his ear, as with bowed head and grave face he walked to and fro, buried in his thoughts.

"Is that you, Warren? You look like a ghost in your whites."

Warren started and looked up, to see Dr. Harrison before him.

"Good evening, Doctor," he said, "will you come into the club and have a peg or——"

"No thanks. Since you have given up coming to me I have come to you. Let's sit down here in the verandah; it's an age since I last had a chat with you."

Warren called his bearer and had chairs placed outside his room. No other Europeans were visible, and the sound of voices and laughter from the billiard-room told them they need fear no interruption for the present.

"Eh, me! I wish my time was up," yawned the Doctor, as he threw himself into his chair. "Thank heaven, I'm near the end of it now! I'll see my girl happily settled and be off. I'm sincerely glad to think she is well provided for. You haven't seen Bairdsley, I think, have you, Warren?"

"No," answered the other.

"He is a nice gentlemanly fellow. No great ability, but a man whom I hope and believe will make her a good husband. He is devoted to her, and she simply worships him."

Warren heard the words with a feeling he could not have described to himself. They told him that he might safely renew his intercourse with Kate, without thought of that danger he believed to exist. If she had given her love in all its entirety, she could have none for himself; she was not the woman to give her heart and lightly withdraw it. The absent lover was secure in such affection as hers.

"I shall like to see Bairdsley," he said, in the most even tone he could command. He could not trust himself to speak of Kate.

"I don't think he will be down in the plains again much before November. He was fairly knocked over by that spell of fever he had in July, and was a wreck when he went away."

"I wonder he did not go home."

"They wanted to send him, but he had only just become engaged to Kate, and the idea of parting with her weighed upon his mind so heavily that the order was rescinded in favour of Simla. I was glad of it for her sake."

"Yes, no doubt it would have been a severe trial to Miss Harrison," said Warren, scarcely knowing what he said.

"Well, we seem to have done what was best in letting him remain in India. He picked up wonderfully soon, and these after-attacks of fever were to be expected whereever he went."

"They are to be married this winter, are they not?" asked Warren, for the sake of saying something.

"Yes; Bairdsley was anxious to carry her off at once, but Kate did not wish it. I was just as well pleased that they should not be in a hurry. Kate is young, and I thought it well that she should have plenty of time to think over the step."

"Had they not known each other long, then?"

"Only a couple of months; Kate is, as you may have thought, old for her years, but I dreaded the possibility of her mistaking her feelings towards him. Bairdsley is one of those men whose attentions are apt to dazzle a young girl."

Warren made no answer; he scarcely dared acknowledge to himself that a ray of dishonourable hope flashed across him.

"So far, I have no reason to think that she was misled by her feelings," continued the Doctor. "The longer he remains away, the more frequently she writes to him. My sister Agnes thought they might have married at once; and Kate certainly seems to have made up her mind thoroughly."

The conversation turned into other channels before long, and after an hour or two, Dr. Harrison took his leave.

"Come and dine quietly with us to-morrow night, Warren," he said, as he rose from his chair. "Kate and I sit like a couple of dummies from the time we sit down until dinner is over. No one else will be there besides ourselves, so you can come in your white kit, and be comfortable."

"Thank you, Doctor. I'll come with pleasure."

"A quarter to eight, you know. Good night."

He went away, and Warren once more sat down to ponder over his position. It was clearer and more simple now. It seemed obvious that Kate Harrison had no thought for any man but this George Bairdsley, and that he had been making a mountain out of a molehill. He had taken upon himself to assume that she was unconsciously falling in love with him, because, forsooth, he had lost his heart to her. It was a piece of egregious vanity on his part, and nothing more: the wish had fathered the thought. However, it was plain enough now; he might go as often as he pleased to the bungalow, for no one but himself was in the least likely to suffer by it if he went three times a day. He had great powers of self-control, and had confidence in his ability to hide his true feeling from her. She need never know that he loved her; indeed, if he wished to keep the friendship of both father and daughter, he must dissemble. If he found that he could not restrain himself; if anything should lead him accidentally to reveal his affection, he would pack up his traps and be off to some other station, out of the way, until she was married and had gone home with her husband.

"Did you see Mr. Warren this evening, papa," asked Kate, when Dr. Harrison returned to the bungalow.

"Yes; we had a long chat. I have asked him to come over to dinner to-morrow."

"I'm very glad we have made it up with him. There are so few really nice men in the station now."

She said nothing more on the subject, but somehow the prospect of having Mr. Warren at dinner gave her an amount of quiet satisfaction which surprised herself; and late as it was, she sent her ayah to summon the cook in order that she might give him fresh orders for the next evening's meal. Mr. Warren was not difficult to please in gastronomical matters. When he grew interested in any topic of conversation he never noticed what was on his plate, and, if he ate it at all, did so mechanically. But Kate took a pride in the table appointments, and her aunt's lessons in housekeeping had not been thrown away. Mr. Warren had once paid her a compliment on the beauty of her floral decorations, which he considered more tasteful than any he had seen in Meerut, and Kate remembered his pretty speech now. It struck her that she did not generally bear such sayings in mind, but accounted for it by the fact that no one was less addicted to them than Mr. Warren, and that this one about the flowers was the only one he had ever made. She quite astonished the bobachee by her liberality in ordering flowers. There were plenty to be had in the bazaar just now, and that functionary was directed to bring the best he could get, regardless of the "much eggpenze," which he assured her would be entailed. He was a very honest cook, as native cooks go, but he retired to his charpoy after the interview, calculating that he could reap at least nine or ten annas "dastouri" from the Missie Baba's love of flowers alone next day. Quite a respectable haul for a man on ten rupees a month, whose expenditure was so rigorously scrutinised that he couldn't even stick on an extra six pie for ghee occasionally without being brought to book for it. The Missie Baba was not half so severe as Brent Memsahib. Perquisites were a mere name during her reign; a poor black man could not take a mouthful of sugar without being found out; and the kerosene oil had been so carefully locked up and measured out, that it took the mesalh chee a fortnight to save up a small bottleful. And even then he had to sell it to the syces belonging to the next bungalow, and burn cocoanut oil himself, for fear of being caught, and getting his tulbub cut for stealing. Brent Memsahib was very just, but she was too strict altogether. English people never did understand the sacredness of Indian customs; only a week ago Major Wilton Sahib had taken his bearer to court for smoking his cheroots, and the bearer had been sent to jail for three days. Really, things had come to a pretty pass if a poor man could not take a few cheroots from master's box without being sent to jail for it. Dr. Harrison Sahib was very good in that way; he never locked up his tobacco, and his bearer respected his confidence, and never by any chance took more than one cigar a day. It was the dastour, the custom; much older than these English laws, and therefore entitled to more respect. However, the bobachee's mild indignation was softened by the thought that there was to be a big bazaar bill next day, and that if he was judicious he might make fourteen annas or even a rupee for himself. He was therefore disposed to be lenient towards the English to-night, and, after reflecting that the sahib who was coming to dinner must be a Burra Sahib whom the Missie Baba delighted to honour, rolled himself up in his cotton cloth from head to heel, and slept the sleep which popular fiction considers the peculiar privilege of the just.

*(To be continued.)*

